# Combination of low-dose rituximab, bortezomib and dexamethasone for the treatment of autoimmune hemolytic anemia

**DOI:** 10.1097/MD.0000000000028679

**Published:** 2022-01-28

**Authors:** Mingkang Yao, Jingjing Zhang, Ying Li, Linlin Lv, Lu Jia, Chunyan Yang, Yu Huang, Haihui Liu, Jian Wang, Mingtai Chen, Hao Zhang

**Affiliations:** a Department of Clinical Medicine, Jining Medical University, Jining, Shandong Province, China; b Department of Hematology, Affiliated Hospital of Jining Medical University, Jining, Shandong Province, China; c Institute of Blood and Marrow Transplantation, Jining Medical University, Jining, Shandong Province, China; d Medical Research Center, Affiliated Hospital of Jining Medical University, Jining, Shandong Province, China.

**Keywords:** bortezomib, dexamethasone, low-dose rituximab, low-dose rituximab, bortezomib and dexamethasone, warm autoimmune hemolytic anemia

## Abstract

Autoimmune hemolytic anemia (AIHA) therapy may be associated with severe complications such as diabetes, hypertension, obesity, osteoporosis, peptic ulcers, infection, and some other diseases. To reduce those effects, we used low-dose rituximab, bortezomib and dexamethasone (LowR-BD regimen) to treat AIHA. The purpose of this study was to evaluate the efficacy and safety of this regimen.

Seven patients with warm AIHA (wAIHA) admitted from March 2020 to October 2020 were treated with LowR-BD regimen: Rituximab 100 mg by intravenous infusion on day 1 combined with bortezomib 1.3 mg/m^2^ by subcutaneous injection on day 2 plus dexamethasone 20 mg by intravenous infusion on days 2, 3. Clinical efficacy and safety were assessed at the regular reexamination of relevant indicators and follow-up.

After 4 cycles of the LowR-BD regimen, the overall response rate (ORR) was 85.71% with a complete response (CR) of 28.57% and a partial response (PR) of 57.14%. After a median follow-up of 12 (range 7–13) months, 5 patients achieved CR and 2 patients had PR status, including 1 patient who did not respond to LowR-BD treatment and reached CR after using methylprednisolone combined with cyclophosphamide. One patient relapsed and achieved PR after retreatment of 2 cycles LowR-BD regimen. The patients tolerated the treatment well and did not complain of apparently adverse reactions except a patient with Sjogren's syndrome and bronchiectasis who developed a severe infection during treatment.

Low-dose rituximab combined with bortezomib and dexamethasone is effective and relatively safe in patients with wAIHA.

## 1 Introduction

Glucocorticoids (GCs) are the first-line treatment for patients with warm autoimmune hemolytic anemia (wAIHA), resulting in a 75% to 80% effective rate. However, some patients are prone to relapse after medication withdrawal and need long-term use which often induce severe complications, including diabetes, hypertension, obesity, osteoporosis, peptic ulcer, infection, etc. The refractory/relapsed patients with steroid-dependency may require second-line regimens such as rituximab, splenectomy, or even third-line regimens such as cyclosporine and cyclophosphamide. However, the efficacy of those treatments are still limited accompanied with many adverse reactions.^[1–3]^ B cell receptor (BCR) can drive B lymphocytes activation and promote their subsequent differentiation into antibody-secreting plasma cells and memory B cells.^[2]^ Rituximab can deplete B cells and prevent existing B cells from maturing into plasma cells but has no significant effect on plasma cells that do not express CD20.^[4]^ In recent years, due to its ability to induce plasma cell apoptosis,^[5–7]^ proteasome inhibitor bortezomib has been used alone or in combination with dexamethasone, rituximab, plasma exchange and other drugs to treat autoimmune diseases such as AIHA, Evans syndrome and immune thrombocytopenia.^[8–13]^ The standard dose of rituximab is 375 mg/m^2^ per week for 4 weeks. Still, it has been reported that low-dose rituximab 100 mg/d has a similar effect,^[14]^ reducing the financial burden of patients and also the adverse reactions. Here in this study, we aim to explore the efficacy of using the low-dose rituximab combined with bortezomib and dexamethasone (LowR-BD) to treat wAIHA.

## 2 Patients and methods

### 2.1 Patients

The clinical data of 7 patients with wAIHA treated with a LowR-BD regimen in the affiliated Hospital of Jining Medical University from March 2020 to October 2020 were retrospectively analyzed. The viral panel including Hepatitis A, B, C, and HIV, chest CT and/or X-ray and heart ultrasonography were done for all patients. Patients with active viral hepatitis, severe immunodeficiency and pregnance or lactation were not included in the study.

### 2.2 Treatment

The LowR-BD regimen was given as 4 7-day cycles. 100 mg Rituximab was injected intravenously on the first day and bortezomib was injected subcutaneously at a dose of 1.3 mg/m^2^ on the second day plus 20 mg dexamethasone by intravenous infusion on the 2nd and 3rd days. At 30 minutes before rituximab injection, patients received 5 mg dexamethasone administered intravenously and 25 mg promethazine hydrochloride injected subcutaneously, followed by 40 mg methylprednisolone through intravenous infusion from another venous pathway during the rituximab injection to prevent allergic reactions. Once the patient developed severe anemia, it is up to clinicians to decide whether they need a blood transfusion or plasma exchange or intravenous infusion of immunoglobulin. 20 to 40 mg/day prednisone was given to the patient after LowR-BD regimen. HGB and reticulocyte ratio were examined per week. If stable, an incremental taper can begin,^[1]^ reducing 5 to 10 mg every week. The blood biochemistry, reticulocyte ratio, and blood routine were closely examined to evaluate the effectiveness and adverse reactions.

### 2.3 Efficacy evaluation

According to the consensus of experts on the diagnosis and treatment of AIHA,^[1,15]^ the efficacy was divided into Complete Response (CR): absence of clinical symptoms, Hb level >110 g/L(female) or 120 g/L(male), no continuous hemolytic features (including normal binding globin level, reticulocyte ratio, serum bilirubin level), direct and indirect Coombs test turned negative or remained positive, but the titer was significantly lower than that before treatment; Partial Response (PR): clinical symptoms disappeared, HGB level >80 g/L, reticulocyte(Ret) ratio <0.05, Serum total bilirubin ≤34.2 umol/L, Coombs test was negative or still positive, but the titer was significantly lower than that before treatment; No response: After treatment, there were still different degrees of anemia or hemolysis, and the laboratory examination did not meet the partial remission criteria.

## 3 Results

### 3.1 Clinical characteristics of patients

Characteristics of the patients included in this retrospective study are summarized in Table 1. Among the 7 patients with wAIHA, 3 were male and 4 were female. The median age was 61 years (range, 39–74 years). Four patients were diagnosed as secondary wAIHA: 1 case was Evans syndrome and systemic lupus erythematous, 1 case was rheumatoid arthritis and Monoclonal B-cell lymphocytosis, 1 case was Waldenstrom macroglobulinemia and 1 case was Sjogren syndrome. A newly diagnosed secondary wAIHA patient 3, who suffers from rheumatoid arthritis and Monoclonal B-cell lymphocytosis, did not respond to the standard dose of GCs for 1 month. And the hemolysis of a primary wAIHA patient 5 aggravated after the reduction of GCs. The rest of the revisited patients also received GCs, rituximab, splenectomy, cyclophosphamide, cyclosporine, mycophenolate mofetil and so on. Before treatment with the LowR-BD regimen, the median haemoglobin level of patients was 54 (47–64) g/L, the median red blood cell count was 1.76 (1.05–2.23) × 10^12^/L, and the median levels of Ret ratio was 10.83% (2.45%–23.22%). In addition, 3 patients received a total of 34 units of red blood cells during previous treatment, with an median of 4 (range 2–16) units per patient.

**Table 1:** Characteristics and outcome of treatment with LowR-BD in patients with wAIHA.

Patient	Sex	Age (years)	Diagnosis	Disease duration	Previous treatment	HGB (g/L) before treatment	RBC (∗10^12^/L) before treatment	Ret (%) before treatment	Transfusion during previous treatment (U)	Days to elevation of HGB > 20 g/L	LowR-BD treatment cycle	Transfusion during LowR-BD treatment (U)	Response after LowR-BD	Time to PR (days)	Time to CR (days)	Follow-up
1	F	65	Evans syndrome, SLE, wAIHA	18 mo	GCs	64	2.23	5.03	0	10	4	2	PR	10	24	CCR
2	F	61	wAIHA	2 mo	FD, PE	63	1.76	10.83	0	9	4	4	CR	9	11	CCR
3	F	48	RA, MBL, wAIHA	1 mo	GCs	54	1.86	8.61	0	8	4	4	CR	8	28	CCR
4	M	39	wAIHA	2 d	FD, PE	58	1.82	13.32	0	7	4	4	CR	7	15	CCR
5	M	74	wAIHA	15 d	GCs	50	1.15	23.22	4	23	4	0	NR	95	233	Reached CR after using CYC for 6. 5 mo
6	F	48	wAIHA, WM	6 yr	COP, Spl, GCs, Rtx, PE, CYC	47	1.08	14.69	18	14	4	4	CR	14	21	Relapse 10 mo later, PR was achieved after treatment with 2 cycles of LowR-BD.
7	M	70	wAIHA, SS	9 mo	GCs, HCQ, TGs, PE, CsA, Rtx, MMF	47	1.05	7.61	12	18	3	12 + 4	PR	26	–	PR

CCR = continuous complete response, COP = cyclophosphamide + vincristine + prednisone, CR = complete response, CsA = ciclosporin, CYC = cyclophosphamide, F = female, FD = first diagnosis, GCs = glucocorticoids, HCQ = hydroxychloroquine, HGB = haemoglobin, LowR-BD = rituximab + bortezomib + dexamethasone, M = male, MBL = Monoclonal B-cell lymphocytosis, MMF = mycophenolate mofetil, PE = plasma exchange, PR = partial response, RA = rheumatoid arthritis, RBC = red blood cell, Ret = reticulocyte, Rtx = rituximab, SLE = systemic lupus erythematous, Spl = splenectomy, SS = Sjogrensyndrome, TGs = Tripterygium glycosides, wAIHA = warm autoimmune hemolytic anemia, WM = waldenstrom macroglobulinaemia.

### 3.2 Therapeutic response

The median days for HGB increase >20 g/L and transfusion independence were 10 (range, 7–23) days. The overall response rate (ORR) after 4 cycles of the LowR-BD regimen was 85.71% (2PR + 4CR), and the median response time for patients who achieved PR was 10 (range 7–95) days. Finally, 6 patients reached complete remission (CR), and the median time to CR was 22.5 (range, 11–233) days, the median haemoglobin level of patients was 117 (112–125) g/L, the median red blood cell count was 3.53 (3.44–3.89) × 10^12^/L, and the median levels of Ret ratio was 1.39% (1.28%–1.47%). Overall, 6 patients received red blood cell transfusions with a median of 4 (range 2–16) units per patient during the 4 consecutive treatment cycles and a total of 34 units of red blood cells. Patient 7 was complicated with bronchiectasis and Sjogren's syndrome and received long-term corticosteroid and immunosuppressive therapy. Before the third cycle treatment of LowR-BD regimen, the patient developed symptoms of acute bronchopulmonary infection, such as fever (maximum body temperature was 40.0°C), cough, purulent sputum, elevated c-reactive protein (CRP) and increased inflammation markers. According to etiological indicators, imipenem and cilastatin sodium (500 mg q6 hours) combined with vancomycin (1.0 g q12 hours) and voriconazole (200 mg q12 hours) were given as anti-infective therapy. After the patient's condition improved, he continued to receive the third cycle of LowR-BD treatment and achieved PR after only 3 cycles. In order to reduce the recurrence of bronchopulmonary infection, the fourth course of treatment was not continued, and PR was maintained during follow-up.

### 3.3 Follow-up

After a median follow-up of 12 (range 7–13) months, 5 cases were in CR status and 2 cases in PR status. After 4 cycles of LowR-BD regimen treatment, the increase of HGB was >20 g/L in patient 5, and the patient was also transfusion independent but exhibited continuous hemolytic features. We have given him plasma exchange combined with methylprednisolone tablet (16 mg qd) and cyclophosphamide tablet (100 mg qd) to control hemolysis (the dosage of methylprednisolone was gradually reduced). A complete response (CR) was achieved after 6.5 months of therapy with cyclophosphamide, and now the CR status was maintained by the patient. Patient 6 relapsed after reaching CR and re-evaluation performed after another 2 cycles of LowR-BD treatment was PR.

### 3.4 Safety

Patient 7 developed an acute bronchopulmonary infection, as previously described. One patient had unstable angina pectoris during the LowR-BD treatment, and the symptoms were relieved after medication given by the department of cardiology, and no related adverse reactions occurred again. One patient developed a hypersensitivity reaction (precardiac discomfort, chest tightness, palpitation, nausea and bloating) during the first infusion of rituximab that improved after treatment of dexamethasone and diphenhydramine. The other patients were well-tolerated, no obvious peripheral neuropathy, neutropenia, thrombocytopenia, and acute liver and kidney failure were observed.

## 4 Discussion

In our study, the overall response rate (ORR) after 4 cycles of the LowR-BD regimen was 85.71% (2PR + 4CR), the median days to HGB increase>20 g/L and transfusion independence were 10 (range, 7–23) days. Except for patient 5, who did not reach CR after 4 cycles of LowR-B, the median time to CR was 22.5 (range, 11–233) days. The data was similar to that reported by Chen et al,^[9]^ who used 500 mg Rtx combined with bortezomib to treat relapsed wAIHA and showed that the median days to HGB increase >20 g/L and transfusion independence were 14 days (range, 5–21). In the study of bortezomib combined with dexamethasone regimen,^[10]^ 6 of the 8 patients achieved response (3 complete responses and 3 responses) after 6 cycles of treatment. Six patients maintained remission after a median follow-up of 14 (6–36) months. Our LowR-BD regimen can also achieve similar results. A meta-analysis^[16]^ showed that the majority of patients received 4 RTX infuses once a week at 375 mg/m^2^; the ORRs were close to 70% for warm AIHA (79%, 95% CI: 60%–90%, 11 studies, 154 patients); the CR rate was 42% (95% CI: 27%–58%, 11 studies, 154 patients) for warm AIHA, our ORR (85.71%) and CR (57.14%) were similar to those previously reported. In our study, the median time to PR was 22.5 days, shorter than the 3 months in the meta-analysis.^[16]^ A total of 34 units of red blood cell transfusions performed during LowR-BD treatment were not significantly different from that during previous treatment. However, blood transfusion was mainly used around the first treatment cycle; only one patient received blood transfusion prior to the third cycle, which indirectly indicated that our regimen worked quickly.

With a median follow-up of 12 months, 5 (4CR + 1PR) patients presented ongoing response to LowR-BD therapy. Interestingly, the overall response rate was the same as that in Phase 3 multicenter, randomized, double-blind RAIHA study^[17]^ in which the patients received 2 infusions of RTX at a dose of 1000 mg 2 weeks apart. In our study, after 4 cycles of LowR-BD treatment, one patient relapsed after remission, another one showed no response. With further prompt treatment, patients achieved remission again. For recurrent/ refractory patients, recommended therapy plan depends on previous treatment regimens used, response, and tolerability.

In general, side effects in our study were mainly infection and infusion-related reactions; peripheral neuropathy, neutropenia, thrombocytopenia, and acute liver and kidney failure were not observed. Furthermore, lowR-BD regimen used low dose rituximab of 100 mg /week, which was less than the one-time treatment of 500 mg reported by Chen et al^[9]^ or 375 mg/m^2^ weekly for 4 weeks.^[1]^ Which exhibited lower adverse reactions and cost than those reported in the meta-analysis,^[16]^ and can be used as an alternative and promising therapeutic strategy for patients with wAIHA. In our study, 1 patient developing acute bronchopulmonary infection with long-term use of immunosuppressants. So that we suggest that patients with high-risk factors for infection include advanced age, long-term use of immunosuppressive agents, and chronic heart and lung disease, should be comprehensively evaluated before treatment with a LowR-BD regimen so that subsequent treatment can be carried out stably.

Our study only included wAIHA patients, and mixed, IgM-mediated Cold agglutinin disease and atypical types were not involved. However, a review of the literature shows that bortezomib is also effective in the treatment of IgM-mediated Cold agglutinin disease. A phase 2 prospective GIMEMA study^[18]^ showed that the overall response rate and CR rate were 31.6% and 15.8%, respectively, in 19 evaluable patients who received one course of bortezomib (intravenous 1.3 mg/m^2^ on days 1, 4, 8, and 11), with few treatment-related toxicities. Individual patient case reports have also shown that bortezomib successfully cured patients with steroid/rituximab refractory IgM-mediated Cold agglutinin disease.^[19]^ In our study, the efficacy and adverse reactions of the LowR-BD regimen in patients with rheumatism were also acceptable, which provides an alternative therapy for some refractory/recurrent rheumatic patients.

In conclusion, the LowR-BD regimen is an effective treatment with acceptable toxicity in wAIHA, which has the advantages of the high degree of efficacy, rapid onset, long duration of the therapeutic effects as well as the absence of severe adverse effects. Physicians can consider this regimen when treating wAIHA. Our study only involved a small number of cases, so further prospective studies with a large sample size are warranted to confirm the efficacy of LowR-BD.

## Author contributions

**Conceptualization:** Jingjing Zhang, Ying Li, Hao Zhang.

**Data curation:** Mingkang Yao, Jingjing Zhang, Hao Zhang.

**Formal analysis:** Mingkang Yao, Jingjing Zhang, Ying Li, Mingtai Chen, Hao Zhang.

**Investigation:** Mingkang Yao, Ying Li, Linlin Lv, Lu Jia, Chunyan Yang, Yu Huang, Haihui Liu, Jian Wang.

**Methodology:** Mingkang Yao, Jingjing Zhang, Ying Li, Linlin Lv, Lu Jia, Chunyan Yang, Yu Huang, Haihui Liu, Jian Wang.

**Project administration:** Mingkang Yao, Jingjing Zhang, Hao Zhang.

**Resources:** Hao Zhang.

**Software:** Mingkang Yao.

**Supervision:** Ying Li.

**Validation:** Jingjing Zhang, Mingtai Chen, Hao Zhang.

**Visualization:** Jingjing Zhang, Mingtai Chen, Hao Zhang.

**Writing – original draft:** Mingkang Yao, Jingjing Zhang, Mingtai Chen, Hao Zhang.

**Writing – review & editing:** Mingkang Yao, Jingjing Zhang, Mingtai Chen, Hao Zhang.
